# Novel Two-Chamber Method for High-Precision TCR Determination of Current Shunts—Part I

**DOI:** 10.3390/s25103197

**Published:** 2025-05-19

**Authors:** Petar Mostarac, Roman Malarić, Hrvoje Hegeduš, Alan Šala

**Affiliations:** Faculty of Electrical Engineering and Computing, University of Zagreb, 10000 Zagreb, Croatia

**Keywords:** temperature coefficient of resistance (TCR), current shunts, measurement uncertainty of TCR

## Abstract

The temperature coefficient of resistance (TCR) plays a crucial role in ensuring the functional accuracy of systems. This article examines the determination of TCR for precision current shunts and presents a novel two-chamber method. The method uses a two-chamber setup for high-precision temperature control, which ensures a reduction in measurement uncertainty when determining the TCR. The two-chamber method is applicable for resistance ratios from 0.1 to 10. The advantages of the proposed method are the improvement of the stability of the reference shunt and the reduction of the measurement uncertainty, and thus a more accurate determination of the TCR. In Part I, the influence of the individual parameters on the determination of the measurement uncertainty of the measured TCR is analyzed.

## 1. Introduction

The precise knowledge and management of temperature coefficient of resistance (TCR) enables the optimization of the performance of electronic devices. In high-precision measuring devices, for example, even small changes in resistance caused by temperature changes can lead to significant measurement errors. Using materials with a low TCR helps to minimize these errors and ensure accurate measurements. In industrial applications, where devices may be exposed to a wide range of operating temperatures, controlling TCR is critical to device reliability and longevity. TCR is a critical parameter for many electronic components and materials [1,2]. In electrical cables, for example, temperature-related changes in resistance can impair the efficiency of energy transmission. A variety of materials and alloys have been developed to meet specific application requirements, allowing engineers to select the optimal materials for their needs. In summary, understanding and controlling TCR is critical to the design and optimization of electronic devices and components, enabling the achievement of superior performance and reliability in a variety of applications.

In practice, different materials have different TCR values. Copper, for example, which is often used as a conductor in electronic circuits, has a positive TCR value. On the other hand, specialized resistors made from alloys such as manganese have a very low TCR value, making them ideal for applications where high resistance stability is required, regardless of temperature changes. Materials with low TCR are preferred for applications where stability and precision are important, such as precision measuring instruments and sensitive electronic components [3,4].

This paper deals with the determination of the temperature coefficient of resistance (TCR) of precision current shunts and proposes a new precise two-chamber method for determination of current shunt TCRs. In precision current measurement, the TCR of current shunts is an important parameter that has a direct effect on the accuracy of the measurements. This is particularly important in high-precision applications, where even small temperature fluctuations can lead to significant measurement errors. A shunt with an inaccurate TCR value can lead to discrepancies in the current measurement results, as the change in resistance with temperature is not properly considered, resulting in incorrect current calculations. This can be particularly problematic in applications that require precise monitoring of electrical parameters, such as metering, power distribution and battery testing. Therefore, understanding and accurately measuring the TCR of current shunts is essential to ensure reliable performance and minimize measurement uncertainty in these systems. Dealing with TCR variations and their effects should be emphasized in the introduction, as they play a central role in the overall accuracy and reliability of current measurement technologies.

Due to the scope of the work, the overall analysis has been divided into two articles. The first article presents the analysis of the influencing variables and explains all parts of the new method and its advantage over the classical approach to the problem. The aim of this article is to demonstrate the measurement method and equipment for calculating TCR for small and medium resistance cage current shunts at different temperatures. It uses a system of two chambers based on solid-state thermoelectric pumps, a measurement system based on high-resolution digitizers and a graphical interface based on Scikit-learn for data input, analysis and visualization. This system and the measurement method are tested with cage current shunts developed at the Department of Electrotechnical Fundamentals and Measurements at the Faculty of Electrical Engineering and Computer Science, University of Zagreb, and are presented in second part of this paper [5,6,7].

The paper will describe the classical approach to TCR determination and the new method for high-precision TCR determination. The methods will be compared according to all important parameters that define the measurement process. It will be shown that the proposed new method offers the following several advantages: it maintains a constant temperature for the reference shunt, ensuring stable current measurement through the second shunt under analysis, and separate temperature management protects the reference shunt from any environmental influences and risks, allowing it to remain in optimal laboratory conditions at all times. Additionally, the method is as equally easy to use as the classic approach, features a compact measurement setup, and enables on-site measurements.

## 2. Precise Two-Chamber Method for Determination of Current Shunt TCRs

The materials used to manufacture current shunts change their resistance properties when the material temperature changes. The resistivity *ρ* of the material does not depend linearly on the change in temperature. The non-linearity of the property is described by polynomials of a certain order, depending on how large the range is when mathematically modeled. Precise current shunts are made from materials and components that do not exhibit a pronounced non-linearity of properties; hence, the usual linear interpretation of resistance changes as a function of temperature with only one coefficient, the TCR.

The TCR is defined by (1), where $R_{t}$ is the resistance at the temperature $T_{t}$, and $R_{0}\;$is the resistance at the reference temperature $T_{0}$ (usually 23 °C) [8,9]. The resulting TCR allows engineers and scientists to quantify how much the resistance changes with temperature and to optimize materials and component designs for specific applications. The unit for (1) is ppm/K (parts per million per Kelvin).

The TCR (1) for precision current shunts (due to the linearity in certain range of usage) is defined by four variables: $R_{t}$, $T_{t}$, $R_{0}$ and $T_{0}$:

(1) $$TCR=\frac{\left(R_{t}-R_{0}\right)}{R_{0}\;\left(T_{t}-T_{0}\right)}\;10^{6}.$$

To ensure an accurate definition of the TCR, it is necessary to control the stability of the temperature and to accurately measure the temperature and resistance of the current shunt.

The stability of the temperature maintained at 23 °C depends on external and internal factors. External factors are changes in the external temperature of the room in which the insulated chamber is located.

### 2.1. Classic Approach for Defining TCR

**Figure 1:**
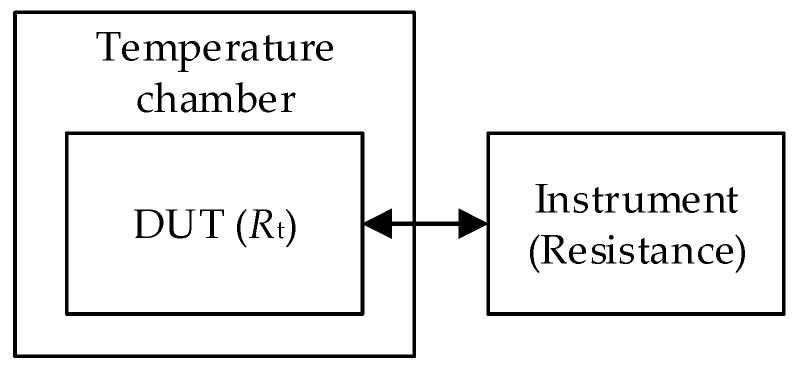
Classic TCR determination setup.

The whole problem can be traditionally solved with one chamber. The system for measuring the TCR is shown in Figure 1 [10].

The system consists of the following:

- a precision temperature chamber that maintains a stable temperature environment for the resistor under test;
- a digital multimeter (DMM) or an equivalent voltage measuring device for the high-precision measurement of resistance at certain temperatures. The DMM should have better temperature characteristics than the DUT (tested resistor $R_{t}$, shunt, element, etc.).

**Figure 2:**
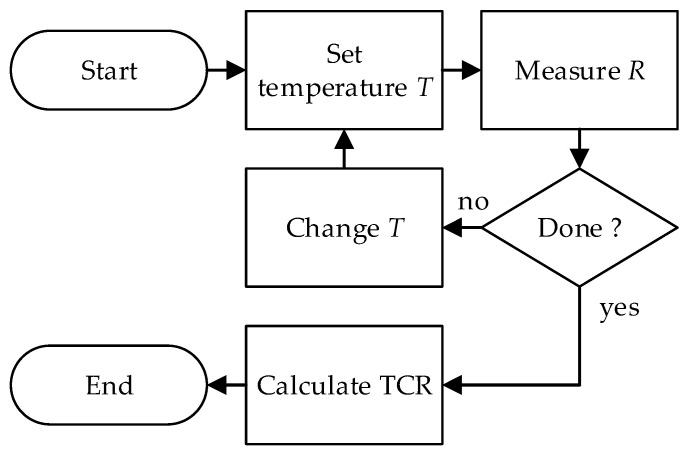
Classic measurement process.

The measurement procedure is relatively linear and easy to carry out (Figure 2):

- set the temperature of the chamber to a specific value. You can start from the highest or lowest temperature in the area of interest and check the humidity once the temperature is stable;
- start the resistance measurement, measure the resistance of the device under test and calculate the TCR as a function of $T_{t}$;
- increase or decrease the temperature and repeat the previous steps. If $R_{0}$ is not known then add a reference temperature in the range of interest.
- calculate the TCR as a function of $T_{t}$.

The classic method assumes that the conditions for temperature stability in the chamber are met and that the measuring device is sufficiently accurate for the method.

### 2.2. High-Precision Approach to Define TCR—Proposed New Method

**Figure 3:**
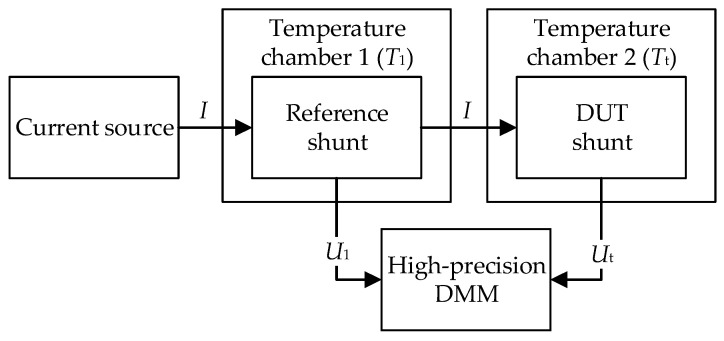
Measurement setup with two chambers.

In this article, a new method is proposed that allows better traceable control of temperature and current through the test specimen, thus ensuring higher precision and lower uncertainties of TCR determination (Figure 3).

The system consists of the following:

- two precision temperature chambers; the first chamber is used to maintain the reference temperature (23 °C) and contains a reference shunt with known $R_{1}$ (calibrated) and the second chamber with $R_{t}$ (DUT), which performs the same task as the classical approach described in the previous subchapter;
- the measurement equipment consists of a reliable current source and a high-precision DMM.

**Figure 4:**
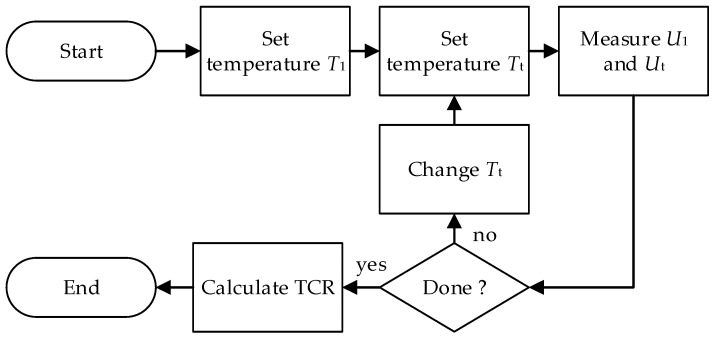
Measurement procedure using the two-chamber method.

The measuring procedure is defined in steps (Figure 4):

- set the temperature in chamber 1 to 23 °C ($T_{1})$;
- set the temperature in chamber 2 to $T_{t}$;
- measure $U_{1}$ and $U_{t}$ using DMM;
- calculate I and $R_{t}$;
- increase or decrease temperature $T_{t}$ of chamber 2 with the values within the temperature range of interest and repeat the previous steps. If $R_{0}$ is not known then add reference temperature in range of interest;
- calculate the TCR as a function of $T_{t}$.

The proposed new method has several advantages. It keeps the temperature of the reference shunt constant and thus ensures a stable determination of the current through the second shunt under investigation. In addition, a separate temperature management system protects the reference shunt from any risk, so that it remains under ideal laboratory conditions at all times. The simple application and compact measurement setup make the method extremely practical. It also offers the possibility of on-site measurement. Two DMMs are used for the simultaneous measurement of $U_{1}$ and $U_{t}$.

## 3. Uncertainty Analysis

In this chapter, the influence of the individual parameters on the determination of the measurement uncertainty of the measured TCR is analyzed. The problem of the vertical asymptote when measuring the TCR at temperatures close to the reference temperature is presented. The problem of the influence of certain measurement uncertainties on the total uncertainty for indirect measurements is presented and compared with the measurement uncertainty for direct measurements (models from Section 2). The main sources of measurement uncertainty in the measurement setup are identified. It is suggested which adjustments to the measurement setup can reduce the measurement uncertainty and which adjustments should not be made. Finally, a detailed analysis with simulations on real examples is given, and a Monte Carlo Simulation is carried out.

### 3.1. The Influence of Uncertainty of Parameters and Variables on the Definition of the TCR

The TCR is defined by four influencing variables (1). A perfect solution for this equation results in stable parametric values for $R_{0}$, $T_{0}$ and $T_{t}$ and with an exact measurement of $R_{t}$. The influence of changed parameters on the TCR can be tested with a partial derivative of (1). The partial derivatives indicate how sensitive the TCR is to the change of certain variables/parameters. If expression (1) is considered as a function of four independent variables, then the partial derivatives (sensitivity coefficients, contributions $c_{i}$) are

(2) $${\left.\frac{\partial TCR}{\partial R_{t}}\right|}_{ppm}=\frac{1}{R_{0}\left(T_{t}-T_{0}\right)}\neq f\left(R_{t}\right),$$

(3) $${\left.\frac{\partial TCR}{\partial R_{0}}\right|}_{ppm}=-\frac{R_{t}}{R_{0}^{2}\;\left(T_{t}-T_{0}\right)}=f\left(\frac{1}{R_{0}^{2}}\right),$$

(4) $${\left.\frac{\partial TCR}{\partial T_{t}}\right|}_{ppm}=-\frac{R_{t}-R_{0}}{R_{0}\;{\left(T_{t}-T_{0}\right)}^{2}}=f\left(\frac{1}{{\left(T_{t}-T_{0}\right)}^{2}}\right),$$

(5) $${\left.\frac{\partial TCR}{\partial T_{0}}\right|}_{ppm}=\frac{R_{t}-R_{0}}{R_{0}\;{\left(T_{t}-T_{0}\right)}^{2}}=f\left(\frac{1}{{\left(T_{t}-T_{0}\right)}^{2}}\right).$$

The partial derivative of TCR varies linearly with the change of $R_{t}$ and is inversely proportional to the square of the change of $R_{0},T_{t}$ and $T_{0}$. Only $R_{t}$ has a linear contribution to TCR, and the partial derivative is constant; it is not a function of $R_{t}$. The other partial derivatives, (3) to (5), are functions of variables to the power of −2. All contributions to TCR decrease with the increase in temperature difference ($T_{t}-T_{0}$), while (4) and (5) decrease with the square of the temperature difference ($T_{t}-T_{0}$). Equations (4) and (5) have opposite signs.

The standard uncertainty for the definition of TCR (1), according to the *Guide to the expression of uncertainty in measurement* (GUM) uncertainty framework [11], can be calculated as follows:

(6) $$u_{c}\left(y\right)=\sqrt{\sum_{i=1}^{N}{\left(c_{i}u\left(x_{i}\right)\right)}^{2}}$$

By applying of expression (6) to expression (1) with usage of partial derivatives as sensitivity coefficients—$c_{i}$ equations (2)–(5) can be calculated as follows:

(7) $$\begin{array}{c} u_{c}\left(TCR\right)=\\ =\sqrt{{\left(\frac{\partial TCR}{\partial R_{t}}\right)}^{2}u^{2}\left(R_{t}\right)+{\left(\frac{\partial TCR}{\partial R_{0}}\right)}^{2}u^{2}\left(R_{0}\right)+{\left(\frac{\partial TCR}{\partial T_{0}}\right)}^{2}u^{2}\left(T_{0}\right)+{\left(\frac{\partial TCR}{\partial T_{t}}\right)}^{2}u^{2}\left(T_{t}\right)} \end{array}$$

The continuation of the analysis will be adapted to more specific parameters and applications of current shunts, as the scope of the general analysis is beyond the scope of this scientific paper.

The usual method for determining the TCR is to measure the resistance of the current shunt at discrete positions of standstill and the specified temperature. Such an approach results in a discrete ∆T whose values change in a certain range around the nominal temperature. The temperature range is defined by applying a current shunt. With stable control of the reference temperature, good insulation materials and precise control of the solid-state thermoelectric pump, the $R_{0}$ can be defined as a parameter with a corresponding uncertainty. Instead of considering four influencing variables, the system can therefore be reduced to two influencing variables, $R_{t}$ and ∆T, where ∆T is defined as follows:

(8) $$∆T=T_{t}-T_{0}.$$

When selecting the temperature range, it should be considered that expression (1) has a vertical asymptote at the value $R_{0}$. Resistance measurements at temperature values close to the reference temperature $T_{0}$ lead to a small resistance change divided by a small temperature change and thus to large fluctuations in the final result of the TCR value. It is the responsibility of the measurement personnel to determine the temperature range of the measurement setup in accordance with the application of the test specimen and considering the vertical asymptote.

### 3.2. Influence of an Inaccurate $R_{t}$ Measurement on the TCR

In the following analysis, the TCR of the current shunt is set to zero, resistance of the shunt is $R_{0}=0.714\;\Omega$, nominal current is equal to 1 A and the referent temperature is $T_{0}=23\;{}^{\circ}C$.

The measuring points $T_{t}$ are in the range from 0 °C to 50 °C with an increment of 1 °C (does not include a temperature of 23 °C).

**Figure 5:**
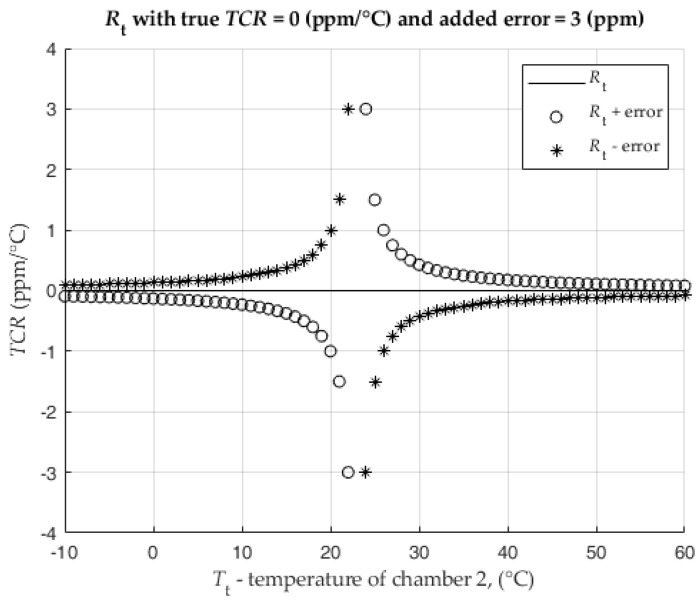
Analysis of the influence of measurement error on the final TCR result.

If the measuring device measures the resistance of $R_{t}$ with an error of ±3 ppm of the nominal resistance value $R_{0}$,

(9) $$R_{t\pm error}=R_{t}\pm R_{0}*\frac{error_{ppm}}{10^{6}},$$

then the final calculated TCR (1) of $R_{t\pm error}$ due to this measurement error (9) is shown in Figure 5. The resulting TCR is presented in comparison to the TCR of $R_{t}$ without error, which is shown in Figure 5 as a line at 0 ppm/°C.

Figure 5 shows that the deviation of the calculated TCR in absolute value increases as $T_{t}$ approaches $T_{0}$. At a temperature difference of 1 °C, the TCR error is equal to the added $R_{t}$ measurement error. The error of TCR decreases asymptotically with the absolute increase of the difference between the measured and the nominal temperature.

### 3.3. Influence of Resistance Ratio—Analysis

When analyzing the influence of the measuring device, i.e., the precision of the measuring device, the authors of the paper analyzed the contribution of classical measuring devices found as standard equipment in metrology and research laboratories. One device that describes the top-class instruments is certainly the DMM manufactured by Keysight under the name 3458A. Other devices from other manufacturers (Fluke, NI, etc.) with similar characteristics (8 and ½ digits) can also be classified in this class [12].

A standard laboratory high-precision DMM Keysight 3458A has the following specifications (type B evaluation):

- for four wire resistance measurements (range: 10 Ω), the stability is (3 + 1) ppm/°C (of Reading + of Range) without autocalibration (ACAL procedure defined from manufacturer) or (1 + 1) ppm/°C with ACAL; accuracy for 24 h and temperature in range $T_{CAL}\pm 1\;{}^{\circ}C$ is (5 + 3) ppm;
- for voltage measurements (range: 1 V), the stability is (1.2 + 0.1) ppm/°C without ACAL and with ACAL is (0.15 + 0.1) ppm/°C; accuracy for 24 h and temperature in range $T_{CAL}\pm 1\;{}^{\circ}C$ is (1.5 + 0.3) ppm.

Maintaining the temperature within the requirements of the equipment manufacturer usually is not a problem and is mostly achieved in laboratory rooms. The main contribution to uncertainty is insufficient accuracy.

If the resistance ($R_{0}=7.14\;\Omega$) is measured directly with the DMM on the range of 10 Ω,the accuracy is 33.57 μΩ. When measuring the voltage drop on the 1 A current shunt, the accuracy is 1.37 μV. One attempt was made to use the observed difference to achieve a lower measurement uncertainty when determining the resistance and TCR of the analyzed shunt by the proposed new method based on indirect measurement (Figure 3).

With a calibrated current shunt and a stable current source, considerable improvement in precision can be achieved. The $R_{t}$ is calculated indirectly using the voltage $U_{t}$ at the tested shunt in chamber 2 and the current through both shunts. The current I is defined as the ratio of the voltage $U_{1}$ at the reference shunt in chamber 1 and the known, calibrated, $R_{1}$. The main contribution of this method is the calculation of the current I for each $T_{t}$ and thus a more stable and accurate determination of $R_{t}$:

(10) $$I=\frac{U_{1}}{R_{1}}\to R_{t}=\frac{U_{t}}{I}\to \;R_{t}=\frac{U_{t}}{U_{1}}R_{1}=U_{t}U_{1}^{-1}R_{1}.$$

The application of expression (6) to the final expression for $R_{t}$ defined in (10) requires calculations of partial derivatives (sensitivity coefficients, contributions $c_{i}$):

(11) $$\begin{array}{c} \frac{\partial R_{t}}{\partial U_{t}}=\frac{R_{1}}{U_{1}},\\ \frac{\partial R_{t}}{\partial R_{1}}=\frac{U_{t}}{U_{1}},\\ \frac{\partial R_{t}}{\partial U_{1}}=-\frac{U_{t}}{U_{1}^{2}}R_{1}. \end{array}$$

The uncertainty of $R_{t}$ by indirect measurement is then calculated by

(12) $$u_{c}\left(R_{t}\right)=\sqrt{{\left(\frac{R_{1}}{U_{1}}\right)}^{2}u^{2}\left(U_{t}\right)+{\left(\frac{U_{t}}{U_{1}}\right)}^{2}u^{2}\left(R_{1}\right)+{\left(-\frac{U_{t}}{U_{1}^{2}}R_{1}\right)}^{2}u^{2}\left(U_{1}\right)}$$

According to the *Guide to the expression of uncertainty in measurement*—GUM (5.1.6., NOTE 2), if the equation has the form

(13) $$y=C\sum_{i=1}^{N}x_{i}^{p_{i}},$$

C stands for a certain constant, and $p_{I}\;$ are known exponents (positive or negative). The relative variance is simply equal to the sum of the estimated relative variances associated with the input variables $x_{i}$ [11]:

(14) $${\left(\frac{u_{C}\left(y\right)}{y}\right)}^{2}=\sum_{i=1}^{N}{\left(\frac{u\left(x_{i}\right)}{x_{i}}\right)}^{2}$$

Equation (14) gives the same value as Equation (12).

If precise determination of $R_{t}$ via the expression (10) has a smaller amount of measurement uncertainty compared to the measurement uncertainty of determining resistance directly by four-wire measurement using a precise DMM (DMM suffix in results), then the proposed method could be used to determine TCR.

The influence of the resistances $R_{1}$ and $R_{0}$, their nominal values on the measurement uncertainty of the novel proposed two-chamber method, was analyzed according to the GUM uncertainty framework (GUM suffix added to result variables) and using the Monte Carlo Simulation/Analysis (results with suffix MCS) [13]. These results are compared to results achieved by the classic approach (DMM suffix in results).

It is necessary to address the issue of effectiveness and usability of the proposed method for different ratios of resistance values of current shunts that are analyzed.

In order to reduce the influence of the calibration precision of the reference resistance $R_{1}$, the value of the measured uncertainty of the laboratory-measured nominal value $R_{1}$ was first set to 1 ppm, and later the analysis for expanded values was made. The value of ~1 ppm is the level that National Metrology Institute achieves when measuring DC resistance values. These data are based on the many years of experience and work of the author of the article on interlaboratory comparisons and everyday calibration work. The voltage and current measurement results were analyzed as if they were measured with a Keysight 3458A, in accordance with the data for the measurement ranges listed in the previous subsection.

The parameters of the preliminary analysis (DMM, GUM and MCS) are listed in Table 1. $N_{MCS}$ is the number of MCS iterations. The DMM accuracy for DC voltage ($V_{DC})$ and resistance (R) is defined as $a_{DMM}{\left(\;\right)}_{rdg}$ and $a_{DMM}{\left(\;\right)}_{rng}$, which are parts of the reading (measured value) and DMM range. $R_{1}$ is the value of the resistance of the reference current shunt. $R_{0}$ are the values of the analyzed shunt resistance at the reference temperature. Maximal current for each $R_{0}$ is given by parameter $I_{N}$. Used current for each ratio is given as I (with respect to maximal allowed dissipation on each current shunt).

The resistance ratio is defined as

(15) $$r=\frac{R_{1}}{R_{0}}\left(\frac{\Omega }{\Omega }\right).$$

The current I is always the nominal value of the current for the shunt with lower nominal current to prevent damage and overheating.

Results of measurement uncertainty measured by GUM, MCS and directly by DMM are given with other data in Table 2. The measurement uncertainty $R_{0}$ and $R_{t}$ are determined using the same proposed method and with usage of the same equipment. The resistance value $R_{0}$ is the value at the reference temperature $T_{0}$. The resistance value of $R_{t}$ is the value at the temperature $T_{t}$. Therefore, the measurement uncertainties $u_{B}(R_{0})$ and $u_{B}(R_{t})$ are identical. In the following, measurement uncertainty (type B) of $R_{t}$ or $R_{0}$ is referred to as $u_{B}(R_{t}$) to make it clear that it is an uncertainty of current shunt (DUT) in chamber 2.

**Figure 6:**
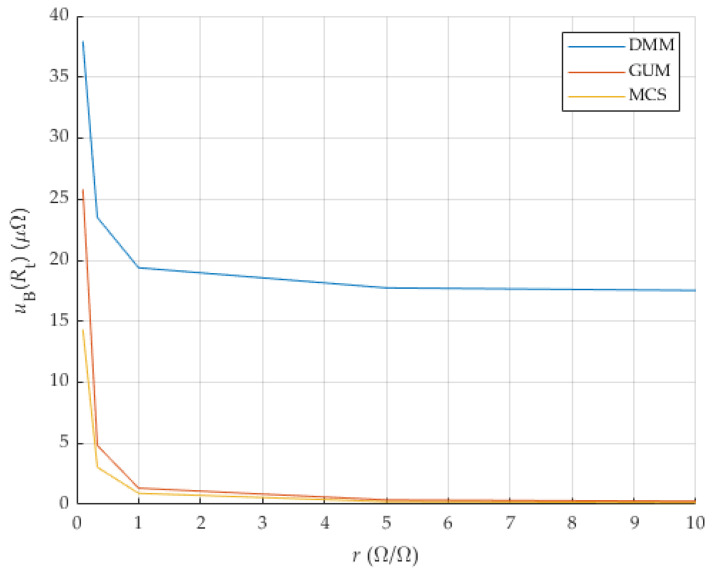
Analysis of the influence of resistance ratio *r* on $u_{B}\left(R_{t}\right)$.

The results in Table 2 show that the proposed method can be used both as a step-down and step-up method, i.e., the referent shunt can have resistance smaller or bigger than the analyzed current shunt. With a bigger resistance ratio, results are better, but in both cases the results are better than in the classic approach with measurement of resistance directly with DMM. Results are also shown in Figure 6. The good applicability of the mentioned method to determine the TCR is ensured, and, therefore, the method presented in this paper can be suitable for a large number of current shunts with different nominal resistances.

The Monte Carlo Simulation achieves slightly better results, which is to be expected, and many authors have addressed similar relationships between MCS and GUM measurement results [14]. Although the GUM modeling approach does not directly define a PDF for the output quantity, it is typically assumed to follow a normal distribution. However, as previously mentioned, the MCS always generates a PDF (probability density function) for the output quantity that aligns with the PDFs of the various input parameters [15].

Additional improvements can be achieved by reduction of the measurement uncertainty (better calibration of the reference shunt) and by using DMM’s transfer accuracy.

In the previous analysis, the resistance ratios and influence of their values were analyzed. Reducing the measurement uncertainty by better calibration (with low expanded calibration of reference shunt $U{\left(R_{1}\right)}_{r}$) and by using the transfer accuracy of the DMM is analyzed in the next subsection.

### 3.4. Influence of the Calibration of the Reference Resistor on the Measurement Uncertainty

This subsection compares the results of the complex measurement uncertainty calculated for the proposed method (Figure 3) according to GUM and MCS, with the results of the measurement accuracy of the directly measured shunt resistance using the classical approach with previously described direct measurement of resistance with DMM (Keysight 3458A) (Figure 1).

**Figure 7:**
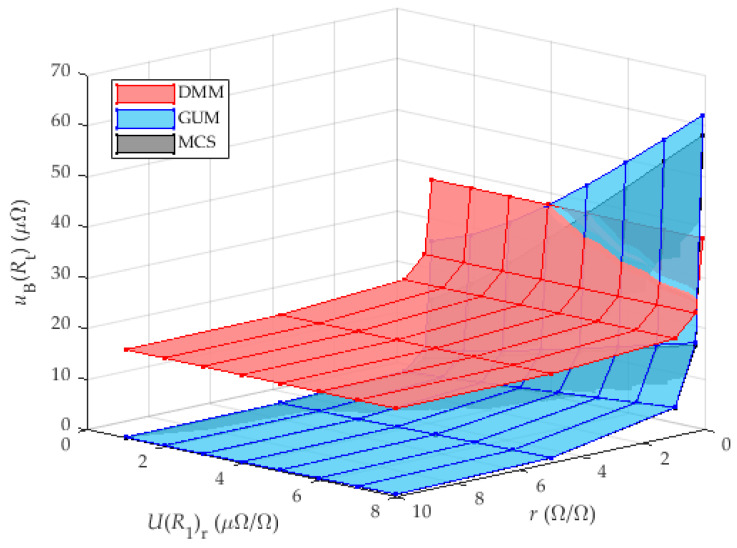
Analysis of the influence of $U{\left(R_{1}\right)}_{r}$ and resistance ratio r on $u_{B}\left(R_{t}\right)$ for DMM with 8 and ½ digits. The results for MCS are very similar to the GUM results and can be seen only partially behind the GUM results.

The ranges for the individual parameters are given in Section 3.3, Table 1. The measurement uncertainty of the calibration of the reference shunt was set to values from 1 ppm to 8 ppm with a 1 ppm step. There is no need to use higher values, as the behavioral trend is also visible at the specified values, and the laboratory calibration usually does not achieve higher values than those specified. In the same chapter, the used accuracies of the DMM for the resistance measurement and the voltage measurement are listed for measurement ranges defined by the size of the shunt resistance and the current through the shunts. Together with the measurement uncertainty of the reference shunt calibration, these parameters define the parameter change ranges for the MCS. The same data were used to calculate the combined measurement uncertainty according to the GUM. The accuracy of the DMM for the resistance measurement generates the value $u_{B}\left(R_{t}\right)-DMM$ (listed in Table 2) and does not depend on $U{\left(R_{1}\right)}_{r}$. The results for GUM, MCS and DMM are shown in Figure 7.

Figure 7 shows the results of the calculation of the measurement uncertainty of the analyzed shunt (DUT) as a function of two variables, the shunt resistance ratio r and the measurement uncertainty of the calibration of the reference shunt $U{\left(R_{1}\right)}_{r}$, which is located in the first chamber. The results obtained by calculating the measurement uncertainty according to GUM achieved higher values than the results obtained by MCS.

The results show a trend towards decreasing measurement uncertainty with increasing accuracy of the laboratory calibration of the reference shunt. In addition, a decrease in measurement uncertainty can be observed with increasing resistance ratio.

The highest values of measurement uncertainty are achieved for a small resistance ratio $R_{1}/R_{0}$. For ratios less than one, the numerator is greater than the denominator in the voltage ratio when calculating the sensitivity coefficient (11), which leads to a greater overall measurement uncertainty.

**Figure 8:**
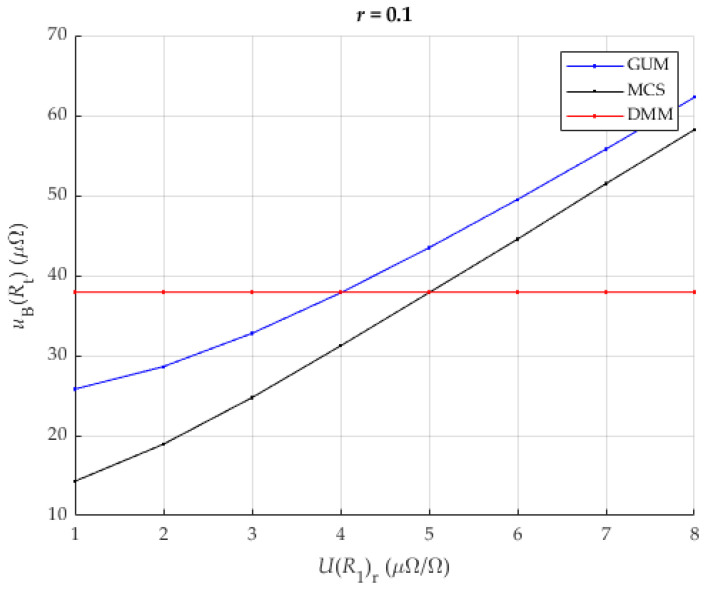
The result of uncertainty of DUT ($R_{t}$) for r=0.1 and different values of $U{\left(R_{1}\right)}_{r}$ for DMM with 8 and ½ digits with both methods: the classical approach (DMM) and the new proposed method (GUM, MCS).

It can be seen that the proposed method in this case provides comparable or better results than the direct measurement of the resistance of the analyzed shunt when the measurement of the reference shunt has a value of less than 4 $\frac{\mu \Omega }{\Omega }$. A more detailed view of Figure 7, focusing on the worst results (ratio r = 0.1), is shown in Figure 8.

### 3.5. Improving DMM Accuracy by Using the Transfer Accuracy

**Figure 9:**
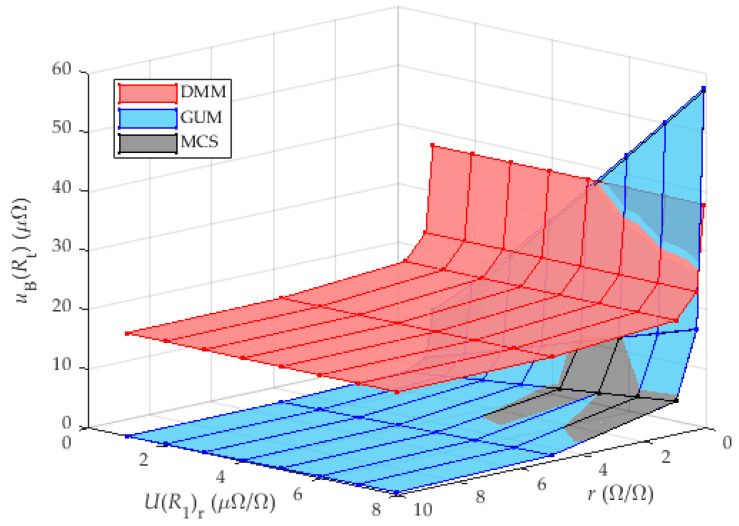
Analysis of the influence of $U{\left(R_{1}\right)}_{r}$ and resistance ratio r on the $u_{B}\left(R_{t}\right)$ with transfer accuracy for DMM with 8 and ½ digits.

The transfer accuracy of the Keysight DMM 3458A for the range of 1 V is 1.5 ppm of the measured value + 3 ppm of the range. The aim of this analysis is to show that the combination of the two conditions, more accurate calibration and use of transfer accuracy, together contributes to a large improvement in the reduction of uncertainty and ensures the applicability of the proposed method under standard laboratory conditions and with classical laboratory equipment (Figure 9).

Figure 8 shows the improvement in the reduction of the measurement uncertainty. The results obtained with GUM are mostly below the measurement uncertainty of the directly measured resistance with the same DMM. The results by MCS also show an improvement. Figure 9 shows an asymptotic decrease in $u_{B}\left(R_{t}\right)$ with an increase in the ratio r. Also, a linear decrease in $u_{B}\left(R_{t}\right)$ with a decrease in $U{\left(R_{1}\right)}_{r}$ can be seen. The results are up to 2.5 times better for higher ratios than the previous analysis. The results show similar trends to the case previously analyzed in Section 3.4.

**Figure 10:**
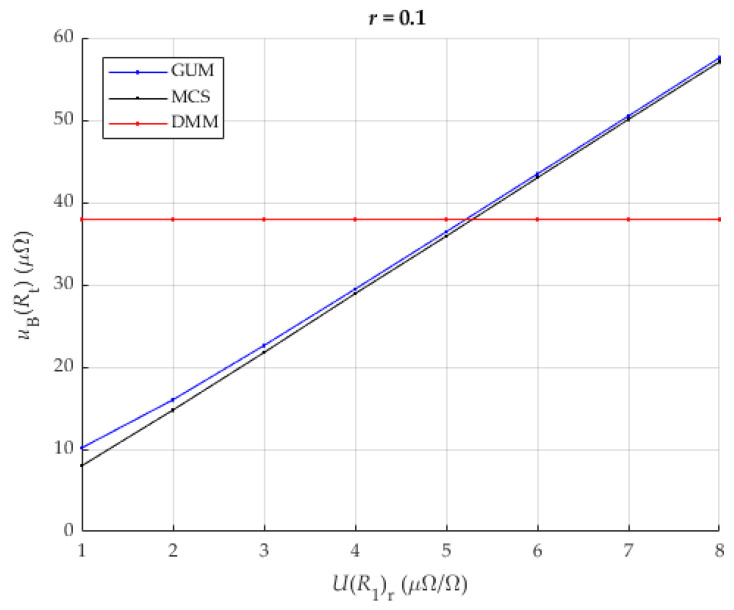
The result of uncertainty of DUT (current shunt—$R_{t}$) for r=0.1 and different values of $U{\left(R_{1}\right)}_{r}$ with transfer accuracy for DMM with 8 and ½ digits.

The largest measurement uncertainties occur at a low resistance ratio r. In this scenario, the proposed method provides results comparable to or better than the direct measurement of the resistance of the analyzed shunt, provided that the reference measurement of the shunt is below 5 μΩ/Ω. A detailed view of Figure 9, showing the least favorable results (with a ratio of r = 0.1), is shown in Figure 10.

If Figure 7 and Figure 9 are compared, then the above-mentioned improvement and the reduction of the measurement uncertainty $u_{B}\left(R_{t}\right)$ can be seen.

For values of $U{\left(R_{1}\right)}_{r}$ smaller than 5 $\frac{\mu \Omega }{\Omega }$, the proposed method gives better results than the direct measurement of the resistance of the current shunt (Figure 10). For larger values of r, the results of the proposed method are better for all values of $U{\left(R_{1}\right)}_{r}$.

The least favorable results from previous analysis (Figure 8 and Figure 10) are used for calculating the propagation of $u_{B}\left(R_{t}\right)$ to the error of TCR determination.

The results show that slightly better results, with lower values, are obtained with the MCS analysis, comparable to the previously cited references comparing GUM and MCS results. In the following, the results according to GUM are used because they define the highest possible values of measurement uncertainty. So, if the method is satisfactory and justifies its use with those results, then it is certain that the final results can only be better.

## 4. Propagation of Resistance Uncertainty to TCR

By combining the results obtained in Section 3, with particular consideration of the influence of the measurement error on the final result of the TCR (Section 3.2) and knowledge of the measurement uncertainty of the method (Section 3.3, Section 3.4 and Section 3.5), it is possible to determine the distribution of the final result of the TCR determination as a function of the temperature $T_{t}$ and $u_{B}\left(R_{t}\right)$.

The process of further reducing the influence of the measurement method (equipment and procedure) on the TCR result can be achieved by avoiding measurements around the reference temperature (23 °C).

### 4.1. Propagation of Measurement Uncertainty

The analysis of the propagation of measurement uncertainty of determination of resistance at a certain temperature to the uncertainty of TCR for the worst case is presented in this subchapter.

The worst case is defined by the highest values of the results (according to GUM due to a bit higher value than MCS, i.e., a worse situation that puts the method to the test), which are comparable to the results of direct resistance measurement with DMM. In this way, the ultimate edge of the applicability of the proposed method is tested.

The analyses were performed according to (2) to (7).

**Figure 11:**
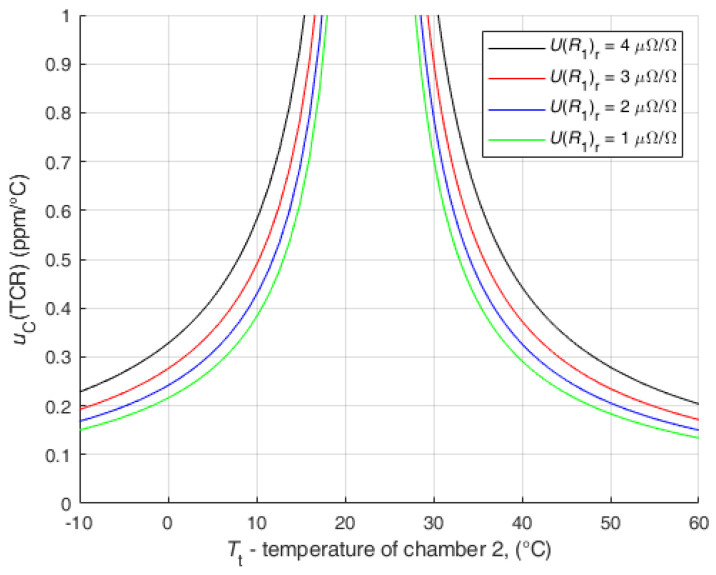
Analysis of the influence of $U{\left(R_{1}\right)}_{r}$ and $u_{B}(R_{t})$ on the $u_{C}(TCR)$ for r=0.1.

For the first analysis, the data obtained for r=0.1 were entered. The comparable results of the proposed method with direct measurement with DMM are obtained for $U{\left(R_{1}\right)}_{r}=4\;\mu \Omega /\Omega$*,* where $u\left(R_{t}\right)=u\left(R_{0}\right)$ = 38 μΩ. The uncertainty of the temperature was set to the value $u\left(T_{0}\right)=u\left(T_{t}\right)=20\;mK$ (according to measurement, which will be presented in next paper). The analysis is shown in Figure 11. All other data, for $U{\left(R_{1}\right)}_{r}<4\;\mu \Omega /\Omega$, with lower $u\left(R_{0}\right)$, are also shown in Figure 11.

To reduce the uncertainty of the TCR below an acceptable value, it is necessary to not measure around the reference temperature in a certain range. For example, to keep the $u_{C}\left(TCR\right)\leq$ 1 ppm/°C for r=0.1 and $U{\left(R_{1}\right)}_{r}=1\;\mu \Omega /\Omega$, the range from 18 °C to 28 °C must be avoided. Outside the range of 10 °C to 33 °C, the $u_{C}\left(TCR\right)$ is less than 0.5 ppm/°C.

Figure 11 is the worst possible result with maximally extended limits of individual parameters to try to determine a limit that is never reached or exceeded in real measurements.

Depending on the measurement requirements and the quality of the reference shunt calibration, it is possible to select a range that fulfils a certain applied accuracy of the method.

**Figure 12:**
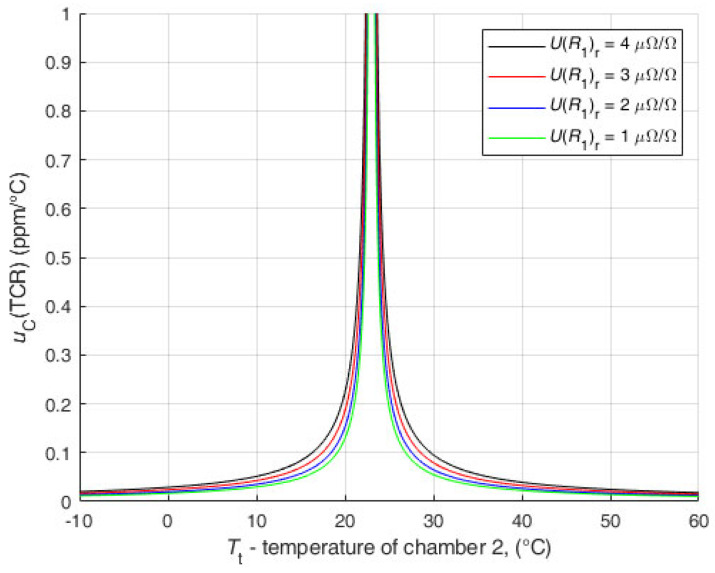
Analysis of the influence of $U{\left(R_{1}\right)}_{r}$ and $u_{B}(R_{t})$ on the $u_{C}(TCR)$ for r=1.

The second analysis (Figure 12) was performed with input data for r=1. For all values of $U{\left(R_{1}\right)}_{r}$, the $u\left(R_{t}\right)$ is much smaller than the uncertainty of the results of the direct resistance measurement with DMM. The values of the TCR uncertainty are even below 1 ppm/°C for temperatures close to the reference temperature.

It can be seen that the results are extremely favorable to the newly proposed method.

### 4.2. Influence of Temperature Measurement Uncertainty

**Figure 13:**
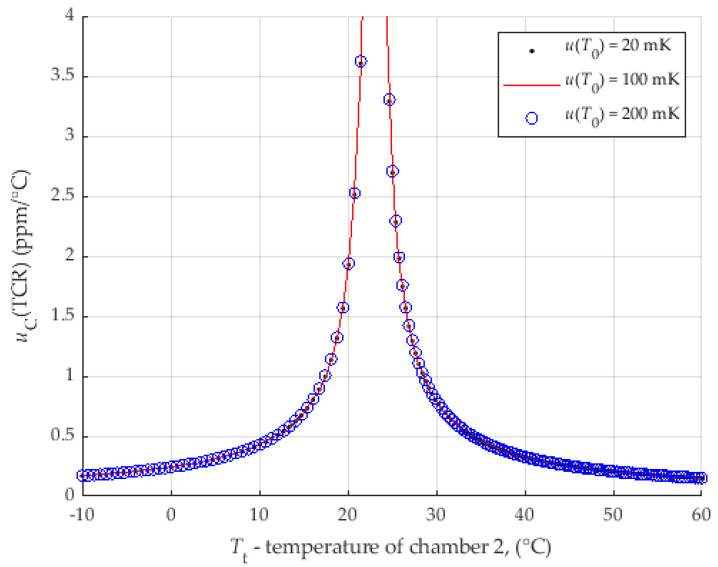
Analysis of the influence of $u\left(T_{0}\right)=u(T_{1})$ on the $u_{C}\left(TCR\right)$ for r=0.1.

The uncertainty of the temperature measurement is not so influential; even with a ten times larger uncertainty, the results are similar. In this analysis, the uncertainty of the temperature was set to the values in a range from $u\left(T_{0}\right)=u\left(T_{t}\right)=20\;mK$ up to $u\left(T_{0}\right)=u\left(T_{t}\right)=200\;mK$. Other data are r=0.1 and $U{\left(R_{1}\right)}_{r}=2\;\mu \Omega /\Omega$. The analysis is shown in Figure 13.

It can be seen that the uncertainty of temperature measurements with values within the normal expected value range (up to a few hundred mK) does not increase the uncertainty budget. The influence of the temperature measurement uncertainty on the uncertainty of the TCR is only noticeable for temperature uncertainty values of more than 1 K, which should never be reached in real measurements.

If the temperature operating conditions of the current shunt (reference temperature) change, previous calibrations and laboratory calibrations can be used to monitor the change and stability of the TCR when the previous TCR values are converted to the new reference temperature.

The procedure for mapping the TCR value to a different reference value can be found in Appendix A.

### 4.3. Application of the Proposed Method

In a typical example of the application of the proposed method, the transfer accuracy of the 8 and ½ digit DMM would be used. In addition, the measurement uncertainty of the calibration of the reference shunt would not be greater than 2 μΩ/Ω with the coverage factor k = 2. The propagated measurement uncertainty $u_{B}(R_{t})$ is then less than or equal to 10 μΩ (Table 3) for all values of the ratio r.

**Figure 14:**
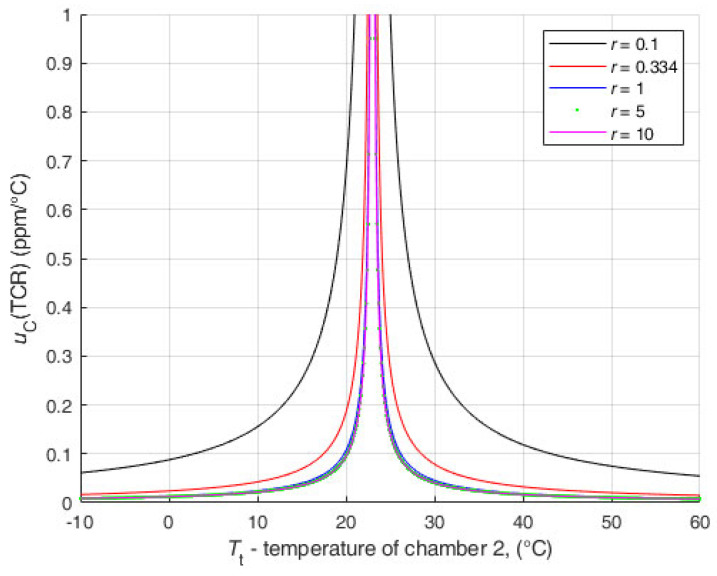
Analysis of the influence of $u_{B}(R_{t})$ and the resistance ratio r on the $u_{C}(TCR)$, with the applied DMM’s transfer accuracy.

The results of the analysis of the influence of $u_{B}(R_{t})$ and r on the final measurement uncertainty of TCR are shown in Figure 14.

The results in Figure 13 show that the new proposed method with the use of transfer accuracy is suitable for determining the TCR with a calibrated reference shunt and indirect measurement of the resistance of the device under test (current shunt). The method is applicable for all resistance ratios r from 0.1 to 10, which is very important for the applicability of the method to a group of shunts (cascade).

## 5. Conclusions

This article explains the classical approach to TCR determination. A new method is proposed that improves the accuracy of TCR determination. The results of the measurement uncertainty analysis using the classical approach and the newly proposed method show an increase in the accuracy using the new method.

The measurement uncertainty analysis was performed for both methods according to GUM in the traditional way using the first-order Taylor approximation and sensitivity coefficients and according to the GUM instructions for the Monte Carlo Simulation (MCS).

The use of an additional chamber to maintain the stability of the reference shunt allows a significant reduction of the measurement uncertainty, first in the determination of the resistance of the shunt for which TCR is analyzed, and then indirectly in the reduction of the measurement uncertainty of TCR.

The proposed method is tested for different resistance ratios of the reference shunt and the shunt for which the TCR is determined. In this paper, the influence of the ratio is analyzed for values ranging from 0.1 to 10. The method is applicable to all ratios, which makes the method suitable for circular comparisons. Circular comparison could further improve the accuracy of the method.

The advantages of the proposed method are that the new method improves the stability of the reference shunt, reduces the measurement uncertainty and enables a more accurate determination of TCR.

The next article (Part II) will describe the experimental setup of the system and its implementation in detail and present the results of the experimental measurements for all the values of the resistance ratio mentioned. In addition, the effects of the accuracy of the temperature measurements in the chambers, the influence of the stability of the current source, the influence of the synchronization of two DMMs and the repeatability of the results will be discussed.

## Figures and Tables

**Table 1:** Parameters of the resistance ratio analysis.

Parameter	Value	Unit	Distribution ^1^/ Comment
$N_{MCS}$	$10^{6}$	Iteration	number of MCS iterations
$a_{DMM}{\left(V_{DC}\right)}_{rdg}$	1.5 (0.3 for transfer accuracy)	$\frac{\mu V}{V}$	rectangular
$a_{DMM}{\left(V_{DC}\right)}_{rng}$	0.3 (0.1 for transfer accuracy)	$\frac{\mu V}{V}$	rectangular
DMM voltage range	1	V	DC
$a_{DMM}{\left(R\right)}_{rdg}$	0.01	$\frac{\mu \Omega }{\Omega }$	rectangular
$a_{DMM}{\left(R\right)}_{rng}$	0.01	$\frac{\mu \Omega }{\Omega }$	rectangular
DMM resistance range	10	Ω	
$R_{1}$	0.714	Ω	
$R_{0}$	(0.0714, 0.143, 0.714, 2.14, 7.14)	Ω	at 23 °C
$I_{N}$	(10, 5, 1, 0.3, 0.1)	A	
I	$\mathrm{Maximal}\;\mathrm{allowed}\;I_{N}$	A	
$U{\left(R_{1}\right)}_{r}\;$ ^1^	(1, 2, 3, 4, 5, 6, 7, 8)	$\frac{\mu \Omega }{\Omega }$	normal distribution
$k_{u\left(R_{1}\right)}\;$ ^2^	1	-	coverage factor

^1^ Expanded calibration uncertainty of reference shunt; relative. ^2^ Set to 1 with the aim to analyze the worst case.

**Table 2:** Results of $u_{B}(R_{t})$ for different current shunt resistance ratios, and expanded uncertainty of referent shunt $U{\left(R_{1}\right)}_{r}$ = 1 ppm.

Parameter	Unit	Values				
r	$\frac{\Omega }{\Omega }$	10	5	1	0.334	0.1
I	A	1	1	1	0.3	0.1
$U\left(R_{1}\right)$	μΩ	0.714	0.714	0.714	0.714	0.714
$u\left(U_{1}\right)$	μV	0.792	0.792	0.792	0.359	0.235
$u\left(U_{t}\right)$	μV	0.235	0.297	0.792	0.729	0.792
$u_{B}\left(R_{t}\right)-GUM$	μΩ	0.258	0.366	1.328	4.830	25.808
$u_{B}\left(R_{t}\right)-MCS$	μΩ	0.143	0.221	0.908	3.043	14.322
$u_{B}\left(R_{t}\right)-DMM$	μΩ	17.527	17.733	19.382	23.498	37.932

**Table 3:** Results of $u_{B}(R_{t}$) for different current shunt resistance ratios, with used transfer accuracy and expanded uncertainty of the referent shunt $U{\left(R_{1}\right)}_{r}$ = 2 ppm (k=2).

Parameter	Unit	Values				
*r*	Ω/Ω	10	5	1	0.334	0.1
$u_{B}\left(R_{t}\right)-GUM$	μΩ	0.1	0.2	0.8	2.4	10.1

## Data Availability

The original contributions presented in this study are included in the article. Further inquiries can be directed to the corresponding author.

## Appendix A

If it is necessary to convert the temperature coefficients of resistance from one reference temperature to another, the following procedure can be used:

In general, the resistance R can be described as a function, Taylor series, of temperature T and *i* temperature coefficients of resistance $\alpha_{rx,i}\;$that are determined in relation to the reference temperature $T_{rx}$:

(A1) $$\begin{array}{c} R\left(T,T_{rx}\right)=R_{r1}\sum_{n=0\;}^{\infty }\alpha_{rx,n}\;{\left(T-T_{rx}\right)}^{n}=\\ \;\;\;\;\;\;=R_{rx}\left(\alpha_{rx,0}{\left(T-T_{rx}\right)}^{0}+\alpha_{rx,1}{\left(T-T_{rx}\right)}^{1}+\cdots +\alpha_{rx,n}{\left(T-T_{rx}\right)}^{n}+\cdots \right), \end{array}$$

where $R_{rx}\;$ is the resistance at the reference temperature $T_{rx}$, and all temperature coefficients at index zero $\alpha_{rx}\left(0\right)$ are equal to one:

(A2) $$\alpha_{rx,0}=1,$$

Usually, $\alpha_{rx,1}\;$is denoted by α (or TCR), $\alpha_{rx,2}$ by β, $\alpha_{rx,3}$ by γ, etc.

For different reference temperatures $T_{r1}\;$and $T_{r2}$, the resistance R at some temperature T must be the same (applying the (A1)) because they are the same function with different reference points:

(A3) $$\begin{array}{c} R\left(T,T_{r1}\right)=R\left(T,\;T_{r2}\right),\;\forall T,\;\\ R_{r1}\sum_{n=0\;}^{\infty }\alpha_{r1,n}\;{\left(T-T_{r1}\right)}^{n}=R_{r2}\sum_{n=0\;}^{\infty }\alpha_{r2,n}\;{\left(T-T_{r2}\right)}^{n}. \end{array}$$

If the change of resistance with temperature is limited to the approximation to term *L*, then (A3) becomes

(A4) $$R\left(T,T_{rx}\right)=R_{rx}\sum_{n=0\;}^{L}\alpha_{rx,n}\;{\left(T-T_{rx}\right)}^{n}+Res.(T,L,T_{rx})$$

where $Res.(T,L,T_{rx})$ is the remainder (residual) modeled as a function of temperature T, limited term L of approximation and reference temperature $T_{rx}$.

When neglecting the residual, expression (A4) becomes finite:

(A5) $$R\left(T,T_{rx}\right)\approx R_{rx}\sum_{n=0\;}^{L}\alpha_{rx,n}\;{\left(T-T_{rx}\right)}^{n}$$

In order to correct the temperature coefficients of the resistance from one reference temperature to another reference temperature, it is necessary to equate the partial derivatives of the Taylor series.

Partial derivatives of (A1) with respect to temperature T are

(A6) ∂kR(T, Trx)∂Tk=Rrx∑n=k∞nkk!αrx,nT−Trxn−k.

When neglecting the residual, expression (A6) becomes finite:

(A7) ∂kR(T, Trx)∂Tk=Rrx∑n=kLnkk!αrx,nT−Trxn−k.

If functions $R\left(T,T_{r1}\right)$ and $R\left(T,T_{r2}\right)$ are identical functions, equal at every point in domain, then their derivatives are also identical.

Applying (A7) to (A3) and equating individual derivatives of $R\left(T,T_{r1}\right)\;$and $R\left(T,\;T_{r2}\right)$ yields the following expressions:

(A8) $$\frac{\partial^{k}R(T,\;T_{r1})}{\partial T^{k}}=\frac{\partial^{k}R(T,\;T_{r2})}{\partial T^{k}},\;k\in \left[1,L\right].$$

For a system of *L* independent equations, *L* partial derivatives of (A8) are required for solving the system of *L* independent equations.

When converting temperature coefficients of resistance from reference temperature $T_{r1}$ to reference temperature $T_{r2}$, coefficients $\alpha_{r1}$ are parameters with known values and coefficients $\alpha_{r2}$ are system variables. Temperature difference $T-T_{rx}$ is marked as $∆T_{rx}$. The system of equations is then

(A9) $$\begin{array}{c} \frac{\partial R(T,\;T_{r1})}{\partial T}=R_{r1}(\alpha_{r1,1}+2\alpha_{r1,2}∆T_{r1}+\cdots +L\alpha_{r1,L}{∆T_{r1}}^{L-1}\\ \;\;\;\;\;\;=R_{r2}(\alpha_{r2,1}+2\alpha_{r2,2}∆T_{r2}+\cdots +L\alpha_{r2,L}{∆T_{r2}}^{L-1}=\frac{\partial R(T,\;T_{r2})}{\partial T}\\ \;\;\;\;\frac{\partial^{2}R(T,\;T_{r1})}{\partial T^{2}}=R_{r1}((2\cdot 1)\alpha_{r1,2}∆T_{r1}+\cdots +L\cdot (L-1)\alpha_{r1,L}{∆T_{r1}}^{L-2}\\ \;\;\;\;\;\;=R_{r2}((2\cdot 1)\alpha_{r2,2}∆T_{r2}+\cdots +L{(L-1)\alpha }_{r2,L}{∆T_{r2}}^{L-2}\\ \;\;\;\;\;\;=\frac{\partial^{2}R(T,\;T_{r2})}{\partial T^{2}}\\ \;\;\;\frac{\partial^{3}R(T,\;T_{r1})}{\partial T^{3}}=R_{r1}((3\cdot 2\cdot 1)\alpha_{r1,3}∆T_{r1}+\cdots +L\cdot (L-1)(L-2)\alpha_{r1,L}{∆T_{r1}}^{L-3}\\ \;\;\;\;\;\;=R_{r2}((2\cdot 1)\alpha_{r2,2}∆T_{r2}+\cdots +L{(L-1)(L-2)\alpha }_{r2,L}{∆T_{r2}}^{L-3}\\ \;\;\;\;\;\;=\frac{\partial^{3}R(T,\;T_{r2})}{\partial T^{3}}\\ \vdots \\ \;\;\;\;\frac{\partial^{L}R(T,\;T_{r1})}{\partial T^{L}}=R_{r1}L!\alpha_{r1,L}{∆T_{r1}}^{L-L}=R_{r2}L!\alpha_{r2,L}{∆T_{r2}}^{L-L}=\frac{\partial^{L}R(T,\;T_{r2})}{\partial T^{L}} \end{array}$$

Every *n*-th derivative cancels the first *n* − 1 elements (orders) of polynomial approximation. As a consequence, the system of Equation (A9) can be written as a system containing the upper triangular matrix.

(A10) $$\begin{array}{c} R_{r2}\left[\begin{array}{ccccc} 1 & 2∆T_{r2} & 3∆T_{r2}^{2} & \cdots & L∆T_{r2}^{L-1}\\ 0 & 2\cdot 1 & 3\cdot 2∆T_{r2} & \cdots & L(L-1)∆T_{r2}^{L-2}\\ 0 & 0 & 3\cdot 2\cdot 1 & \cdots & L(L-1)(L-2)∆T_{r2}^{L-3}\\ \vdots & \vdots & \vdots & \ddots & \vdots \\ 0 & 0 & 0 & 0 & L! \end{array}\right]\left[\begin{array}{c} \begin{array}{c} \alpha_{r2,1}\\ \alpha_{r2,2}\\ \alpha_{r2,3}\\ \vdots \end{array}\\ \alpha_{r2,L} \end{array}\right]=\\ \;\;\;\;\;\;\;=R_{r1}\left[\begin{array}{ccccc} 1 & 2∆T_{r1} & 3∆T_{r1}^{2} & \cdots & L∆T_{r1}^{L-1}\\ 0 & 2\cdot 1 & 3\cdot 2∆T_{r1} & \cdots & L(L-1)∆T_{r1}^{L-2}\\ 0 & 0 & 3\cdot 2\cdot 1 & \cdots & L(L-1)(L-2)∆T_{r1}^{L-3}\\ \vdots & \vdots & \vdots & \ddots & \vdots \\ 0 & 0 & 0 & 0 & L! \end{array}\right]\left[\begin{array}{c} \begin{array}{c} \alpha_{r1,1}\\ \alpha_{r1,2}\\ \alpha_{r1,3} \end{array}\\ \vdots \\ \alpha_{r1,L} \end{array}\right] \end{array}$$

To solve (A10) for exact numerical values, the simplest way is to use the classic approach and calculate the right side of (A10) and find the inverse of the matrix on the left side of the equation.

In order to solve the system symbolically, considering the temperature coefficients of the resistance at the first reference temperature $T_{r1}$, and to obtain applicable formulas for the transformation of the coefficients to the second reference point $T_{r2}$, it is necessary to solve the system symbolically from bottom to top, since the transformation of the last coefficient is clearly defined in the last row of the matrix system. Thus, for the last coefficient $\alpha_{r2,L}$ in the system presented in (A10) (solution for *L* = 1),

(A11) $$\begin{array}{c} R_{r2}L!\alpha_{r2,L}=R_{r1}L!\alpha_{r1,L}\to R_{r2}\alpha_{r2,L}=R_{r1}\alpha_{r1,L}\to \\ \alpha_{r2,L}=\frac{R_{r1}}{R_{r2}}\;\alpha_{r1,L} \end{array}$$

The resulting expression (A11) can be then inserted into the penultimate row, yielding to a transformation expression for the coefficient $\alpha_{r2,L-1}$. The mentioned procedure continues until the expressions for the transformation of all coefficients are calculated.

*Solution for L = 2*

For second order approximation of temperature dependency (*L* = 2) of resistance with a known,

(A12) $$R\left(T,T_{r1}\right)=R_{r1}\left(1+\alpha_{r1,1}∆T_{r1}+\alpha_{r1,2}∆T_{r1}^{2}\right),$$

so that when it is necessary to determine new coefficients defined by a different reference temperature,

(A13) $$R\left(T,T_{r2}\right)=R_{r2}\left(1+\alpha_{r2,1}∆T_{r2}+\alpha_{r2,2}∆T_{r2}^{2}\right),$$

and so by applying the expression (A8) to (A12) and (A13), equating the first and second derivatives of the expression results in a system of two equations with two unknowns (2 × 2):

(A14) $$\begin{array}{c} \frac{\partial R\left(T,\;T_{r2}\right)}{\partial T}=R_{r2}\alpha_{r2,1}+{2R_{r2}\alpha }_{r2,2}∆T_{r2}=R_{r1}\alpha_{r1,1}+{2R_{r1}\alpha }_{r1,2}∆T_{r1}=\frac{\partial R\left(T,\;T_{r1}\right)}{\partial T},\\ \frac{\partial^{2}R(T,\;T_{r2})}{\partial T^{2}}={2R_{r2}\alpha }_{r2,2}={2R_{r1}\alpha }_{r1,2}=\frac{\partial^{2}R(T,\;T_{r1})}{\partial T^{2}}, \end{array}$$

and the solution to the system is

(A15) $$\begin{array}{c} \;\alpha_{r2,2}=\frac{R_{r1}}{R_{r2}}\;\alpha_{r1,2}\\ \;\alpha_{r2,1}=\frac{R_{r1}}{R_{r2}}\left(\alpha_{r1,1}+2\left(T_{r2}-T_{r1}\right)\alpha_{r1,2}\right). \end{array}$$

*Solution for L = 3*

Third order approximations of temperature dependency (*L* = 3) are as follows:

(A16) $$\begin{array}{c} R\left(T,T_{r1}\right)=R_{r1}\left(1+\alpha_{r1,1}∆T_{r1}+\alpha_{r1,2}∆T_{r1}^{2}+\alpha_{r1,3}∆T_{r1}^{3}\right),\\ R\left(T,T_{r2}\right)=R_{r2}\left(1+\alpha_{r2,1}∆T_{r2}+\alpha_{r2,2}∆T_{r2}^{2}+\alpha_{r2,3}∆T_{r2}^{3}\right), \end{array}$$

and derivatives and system of equations (3 × 3) are

(A17) $$\begin{array}{c} \;\frac{\partial R\left(T,\;T_{r2}\right)}{\partial T}=1\cdot R_{r2}\alpha_{r2,1}∆T_{r2}^{0}+{2R_{r2}\alpha }_{r2,2}∆T_{r2}^{1}+{3R_{r2}\alpha }_{r2,3}∆T_{r2}^{2}\\ \;=1\cdot R_{r1}\alpha_{r1,1}∆T_{r1}^{0}+{2R_{r1}\alpha }_{r1,2}∆T_{r1}^{1}+{3R_{r1}\alpha }_{r1,3}∆T_{r1}^{2}\\ \;=\frac{\partial R\left(T,\;T_{r1}\right)}{\partial T},\\ \;\frac{\partial^{2}R(T,\;T_{r2})}{\partial T^{2}}={2\cdot 1R_{r2}\alpha }_{r2,2}{∆T}_{r2}^{0}+{3\cdot 2R_{r2}\alpha }_{r2,3}∆T_{r2}^{1}\\ {\;=2{\cdot 1R}_{r1}\alpha }_{r1,2}∆T_{r1}^{0}+{3\cdot 2R_{r1}\alpha }_{r1,3}∆T_{r1}^{1}=\frac{\partial^{2}R(T,\;T_{r1})}{\partial T^{2}},\\ \frac{\partial^{3}R(T,\;T_{r2})}{\partial T^{3}}={3\cdot 2\cdot 1R_{r2}\alpha }_{r2,3}∆T_{r2}^{0}={3\cdot 2\cdot 1R_{r1}\alpha }_{r1,3}∆T_{r1}^{0}=\frac{\partial^{3}R(T,\;T_{r1})}{\partial T^{3}} \end{array}$$

and the solutions are

(A18) $$\begin{array}{c} \alpha_{r2,3}=\frac{R_{r1}}{R_{r2}}\;\alpha_{r1,3},\\ \alpha_{r2,2}=\frac{R_{r1}}{{\;R}_{r2}}\left(\alpha_{r1,2}+3\left(T_{r2}-T_{r1}\right)\alpha_{r1,3}\right)\\ \alpha_{r2,1}=\frac{R_{r1}}{R_{r2}}\left(\alpha_{r1,1}+2\left(T_{r2}-T_{r1}\right)\alpha_{r1,2}+3{\left(T_{r2}-T_{r1}\right)}^{2}\;\right)\alpha_{r1,3} \end{array}$$
